# Analysis of m^7^G methylation modification patterns and pulmonary vascular immune microenvironment in pulmonary arterial hypertension

**DOI:** 10.3389/fimmu.2022.1014509

**Published:** 2022-12-05

**Authors:** Desheng Wang, Yanfei Mo, Dongfang Zhang, Yang Bai

**Affiliations:** ^1^ Department of Clinical Pharmacology, School of Pharmacy, China Medical University, Shenyang, Liaoning, China; ^2^ Department of Pharmacognosy, School of Pharmacy, China Medical University, Shenyang, Liaoning, China

**Keywords:** pulmonary arterial hypertension, machine learning, immunity, microenvironment, methylation, m^7^G

## Abstract

**Background:**

M^7^G methylation modification plays an important role in cardiovascular disease development. Dysregulation of the immune microenvironment is closely related to the pathogenesis of PAH. However, it is unclear whether m^7^G methylation is involved in the progress of PAH by affecting the immune microenvironment.

**Methods:**

The gene expression profile of PAH was obtained from the GEO database, and the m^7^G regulatory factors were analyzed for differences. Machine learning algorithms were used to screen characteristic genes, including the least absolute shrinkage and selection operator, random forest, and support vector machine recursive feature elimination analysis. Constructed a nomogram model, and receiver operating characteristic was used to evaluate the diagnosis of disease characteristic genes value. Next, we used an unsupervised clustering method to perform consistent clustering analysis on m^7^G differential genes. Used the ssGSEA algorithm to estimate the relationship between the m^7^G regulator in PAH and immune cell infiltration and analyze the correlation with disease-characteristic genes. Finally, the listed drugs were evaluated through the screened signature genes.

**Results:**

We identified 15 kinds of m^7^G differential genes. CYFIP1, EIF4E, and IFIT5 were identified as signature genes by the machine learning algorithm. Meanwhile, two m^7^G molecular subtypes were identified by consensus clustering (cluster A/B). In addition, immune cell infiltration analysis showed that activated CD4 T cells, regulatory T cells, and type 2 T helper cells were upregulated in m^7^G cluster B, CD56 dim natural killer cells, MDSC, and monocyte were upregulated in the m^7^G cluster A. It might be helpful to select Calpain inhibitor I and Everolimus for the treatment of PAH.

**Conclusion:**

Our study identified CYFIP1, EIF4E, and IFIT5 as novel diagnostic biomarkers in PAH. Furthermore, their association with immune cell infiltration may facilitate the development of immune therapy in PAH.

## Graphical Abstract

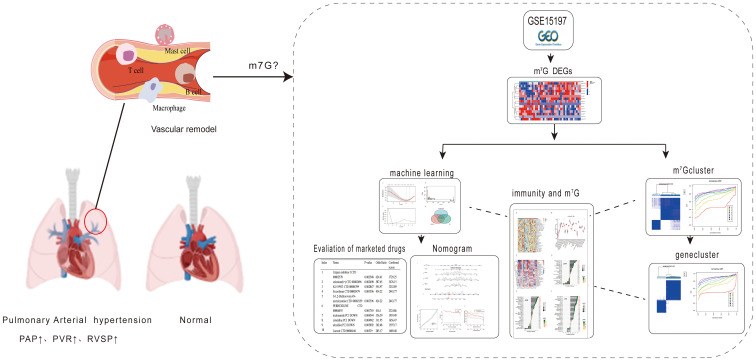

## Introduction

Pulmonary arterial hypertension (PAH) is defined as mean pulmonary arterial pressure ≥ 25 mmHg, pulmonary artery wedge pressure ≤ 15 mmHg, and pulmonary vascular resistance (PVR) > 3 Wood units (1). Patients develop PAH for a variety of reasons, with the right heart and lung disease being the most common cause of pulmonary hypertension (PH) in nearly all regions of the world (2). The pathogenesis of PAH is referred to as a complex interaction between immune cells and vascular stromal cells. *Tanby* et al. have found the presence of anti-endothelial cell antibodies in idiopathic pulmonary arterial hypertension (IPAH), which seems to suggest that the humoral immune system can influence endothelial cell proliferation in PAH (3). B lymphocytes, the cellular basis of the humoral immune response, are less sensitive to monocrotaline (MCT) or hypoxia-induced PAH (HPH) in rats (4). Macrophages are increased in PAH patients and animal models (5). There is an increase in the number of lymphocytes, macrophages, mast cells, and dendritic cells around the pulmonary artery vasculature of PAH patients (5). These suggest that immune dysfunction may play an important role in pulmonary perivascular inflammation and the pathological progression of PAH (6, 7).

RNA methylation is a common epigenetic modification in eukaryotes, including N6-methyladeno-sine (m^6^A), 5-methylcytosine (m^5^C), and 7-methylguanosine (m^7^G). M^7^G acts as a positively charged mRNA 5’ cap modification (8, 9) which is involved in the progress of mRNA transcription (10), mRNA splicing (11) and mRNA translation (12). M^7^G modifications are widespread in tRNA and rRNA (13). It has been implicated in certain diseases, for example, the RNA methyltransferase like 1 (METTL1) catalyzes m^7^G modification of tRNA to drive oncogenic transformation by remodeling the mRNA “Transcriptome” (14). *Zhang* et al. have shown the distribution signature of the internal mRNA m^7^G methylome in human HeLa, HepG2, and HEK293T cell lines (9). M^7^G modifications are also present in internal mRNAs (15), and these internal m^7^G methylations affect RNA function and are implicated in human diseases, such as tumors and immune diseases (16, 17).

Recent studies have shown that m^7^G-related microRNAs have regulatory roles in the tumor micro-environment of clear cell renal cell carcinoma (18). Meanwhile, METTL1 promotes vascular endothelial growth factor A mRNA translation in an m^7^G methylation-dependent manner, and m^7^G in mRNA has been shown to be associated with vascular diseases (19). In addition, m^7^G-related long noncoding RNAs (lncRNAs) may be involved in tumor immunity, such as lung adenocarcinoma (20), skin melanoma (21) and colon cancer (22). Interestingly, m^7^G methylation of lncRNAs has recently been reported to be associated with hypoxic PH (23). The cancer model of “primary pulmonary hypertension” was first proposed by *Voelkel* et al. (24). PAH has many cancer-like pathogenic features and signaling pathways (25, 26). A hallmark of cancer is the infiltration of immune cells (27), which play a key role in tumor progression by creating an inflammatory microenvironment that promotes tumor growth (28). Studies have found that 45-50% of lung cancer patients have elevated pulmonary systolic blood pressure, and pulmonary artery enlargement (29). Similarly, PAH is characterized by perivascular infiltration of innate and adaptive immune cells, including mast cells, macrophages, B cells, and T cells (4, 30). The above suggests that both cancer and PAH appear to actively suppress immune defense mechanisms. Therefore, we have reason to believe that the regulatory effect of m^7^G on tumor immune cells may also occur in PAH. Current research regarding RNA methylation regulation is mostly associated with tumor diseases. Little is known about whether RNA methylation signatures are applicable to PAH and how m^7^G is involved in PAH progression is currently unclear. Therefore, we hypothesized that m^7^G may participate in the process of pulmonary vascular remodeling in PAH by regulating the immune microenvironment.

The purpose of this study is to explore the role of m^7^G methylation modification in the immune microenvironment of PAH through bioinformatics technology and machine learning methods, to further clarify the role of m^7^G regulators in the progression of PAH, and to provide new ideas for PAH treatment.

## Materials and methods

### Workflow overview

First, we downloaded the GSE15197 dataset (31) and the GSE113439 dataset (32) from the Gene Expression Omnibus database (GEO, http://www.ncbi.nlm.nih.gov/geo/). The dataset was batch corrected by the limma package, and the differential analysis of m^7^G regulators was performed. At the same time, the intersection of least absolute shrinkage and selection operator (LASSO) regression curve, random forest (RF) and support vector machine model (SVM-RFE) algorithms were used to screen disease characteristic genes. Then, a nomogram model was constructed and the area under the receiver operating characteristic (ROC) curve was calculated to evaluate the model, performance, and validated on the GSE113439 dataset. In addition, we used the single sample gene set enrichment analysis (ssGSEA) algorithm to quantify the relative abundance of immune cells in PAH, by consensus clustering to classify m^7^G regulators and differential genes of the and m^7^G regulatory factors were analyzed based on subtypes. Finally, we evaluated the marketed drugs through the screened disease signature genes in PAH. See graphical abstract for details.

### Datasets used in this study

Download the GSE15197 and GSE113439 datasets from the GEO database. GSE15197 included lung tissue samples from 26 patients with PAH and 13 Normal, including PAH subjects (n=18), IPF subjects with secondary PH (n=8), and Normal (n=13). The GSE113439 dataset included lung tissue from 15 patients with PAH and 11 Normal, of which the PAH group includes 6 patients with idiopathic PAH, 4 patients with PAH secondary to connective tissue disease (CTD), and 4 patients with congenital PAH. PAH patients with chronic heart disease (CHD) and one patient with chronic thromboembolic pulmonary hypertension (CTEPH).

### Identification of differential genes

We searched and identified m^7^G-related genes from published literature (33, 34), and then extracted and analyzed using the “limma” package in R statistical software (35) GSE15197 data, and screened for differentially expressed m^7^G regulators between PAH patients and Normal.

### Screening of disease signature genes

We used LASSO, RF and SVM-RFE three machine learning algorithms to screen disease characteristic genes. LASSO regression was performed using the “glmnet” R package (36), and 10-fold cross-validation was used to analyze PAH samples and normal samples. Random forest model building using the “random forest” R package (37). In addition, the SVM-RFE model was generated by the“e1071”SVM function, which could be used to determine the number of best-ranked genes (38).

### Construction of the diagnostic model

To predict the incidence of the disease, we built a predictive model by using the “rms” R package. The corresponding genes were scored individually by “Points”, and total genes scores were summarized “Total Points”. The predictive power of the nomogram model was assessed by calibration curves, and the clinical value of the model was evaluated using decision curve analysis (DCA) and clinical impact curve analyses. Furthermore, to further estimate the predictive value of PAH diagnosis, we performed calculations with “pROC package” (39). The larger the value of AUC, the higher the accuracy of the prediction model, which was further validated in the GSE113439 dataset.

### Consensus clustering

Using an unsupervised clustering algorithm, implemented in the “Consensus Cluster Plus” software package (40), the differential genes and 15 kinds of m^7^G regulators of the GSE15197 gene set were clustered to determine their optimal clustering and classes.

### SsGSEA immunoassay

SsGSEA was performed to use the “GSVA” package in Reversion 4.2.1, and the selection of immune cell types were derived from a recent publication by *Charoentong* et al., which stocked various human immune cell subtypes, including activated CD8 T cells, activated dendrites cells, macrophages, natural killer T cells, and regulatory T cells, etc. (41).

### Marketed drug evaluation

It was one of the important contents of this study to evaluate the marketed drugs through the screened disease characteristics. The drug molecules were identified by using the Drug Signatures database (DSigDB) in the Enrichr database (https://maayanlab.cloud/Enrichr/).

### Statistical analysis

All data processing was done in R 4.2.1 software. T-test and Wilcox Test were used in this study, depending on the type of data. The error bars in the figures represented the standard error of the standard deviation (S.D.). Spearman correlation analysis was used to assess the relationship between diagnostic gene expression and infiltrating immune cells. P<0.05 was considered statistically significant.

## Results

### Differential analysis of m^7^G regulators in PAH patients

**Figure 1:**
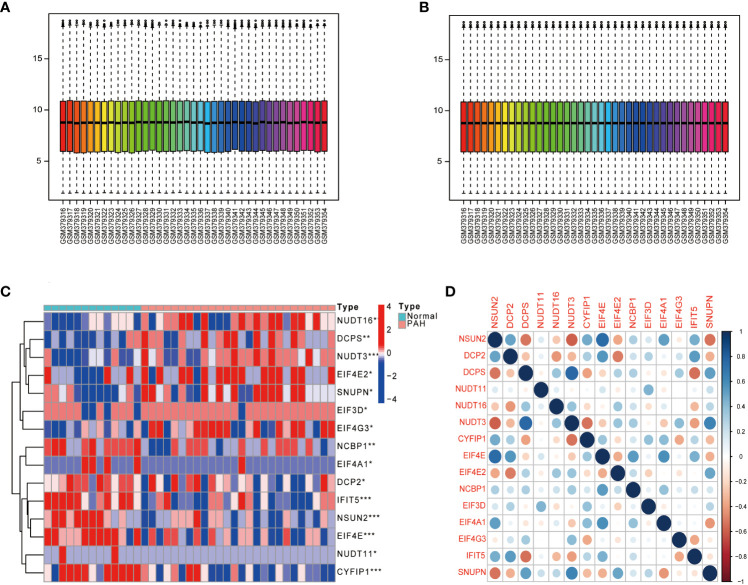
Gene differential expression analysis. **(A)** GSE15197 data before normalization. **(B)** GSE15197 normalized data. **(C)** Differential expression of m^7^G-regulated genes in PAH patients and normal controls. **(D)** Heat map of the correlation of m^7^G genes. Both horizontal and vertical coordinates represent genes, and different colors represent correlation coefficients (blue represents positive correlation and red represents negative correlation in the diagram). *P < 0.05, **P < 0.01 and ***P < 0.001. asterisks (*) stand for significance levels.

The dataset GSE15197 was normalized (**Figures 1A, B**), and m^7^G regulators were differentially analyzed in lung tissue samples from PAH patients and normal using the limma package, as shown in **Figure 1C**, 15 kinds of m^7^G regulators (NSUN2, DCP2, DCPS, NUDT11, NUDT16, NUDT3, CYFIP1, EIF4E, EIF4E2, NCBP1, EIF3D, EIF4A1, EIF4G3, IFIT5, and SNUPN) showed significant differences between PAH patients and Normal. Next, the “Performance Analytics” R software package was used to perform correlation analysis on 15 kinds of m^7^G regulators with significant differences, and it was found that there were different degrees of correlation between the 15 kinds of m^7^G regulators, blue represents positive correlation, red represents negative correlation, the darker the color, the stronger the correlation (**Figure 1D**). The above analysis showed that m^7^G regulators are highly different and correlated between normal and PAH patients, indicating that the expression imbalance of m^7^G regulators played a crucial role in the occurrence and development of PAH.

### Screening of disease characteristic genes

**Figure 2:**
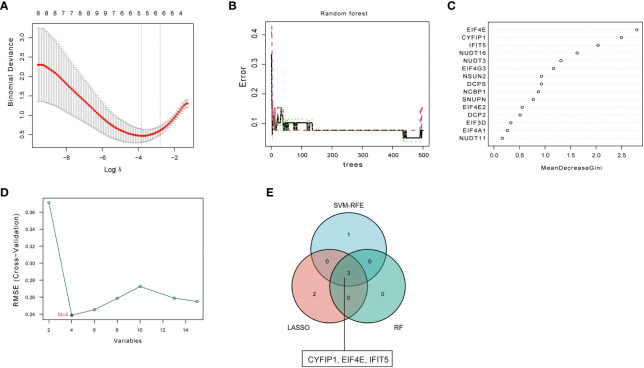
Screening of disease signature genes. **(A)** Selection of the best Log (ʎ) value for LASSO regression. The x-axis represents the Log (ʎ) value, and the y-axis represents the error rate of cross-validation errors. **(B)** The influence of the number of decision trees on the error rate. The x-axis repre- sents the number of decision trees, and they-axis indicates the error rate. **(C)** Results of the Ginico efficient method in random forest classifier. The x-axis indicates the genetic variable, and the y-axis represents the importance index. **(D)** Variation curve of gene cross-validation error in SVM-RFE algorithm. **(E)** Venn diagram showing 3 disease signature genes shared by LASSO, SVM-RFE and RF arithmetic methods.

To screen for disease-characteristic genes, we used three different machine learning algorithms to analyze 15 kinds of m^7^G regulators with significant differences. First, the 15 kinds of m^7^G regulators were filtered through LASSO regression analysis, as shown in **Figure 2A**, when the best Log (ʎ) in the figure is equal to 5, the corresponding cross-validation error rate is the smallest, thus determining NSUN2, CYFIP1, EIF4E, NCBP1, and IFIT5 as signature genes of PAH. Next, the eight genes of the disease were screened by the RF method, and about 120 trees were selected as the parameters of the final model, which showed stable errors in the model (**Figure 2B**), and then, we selected genes with scores greater than 2 (EIF4E, CYFIP1, and IFIT5) for subsequent analysis (**Figure 2C**); The characteristic genes of the disease were determined by the SVM-RFE algorithm, as shown in the **Figure 2D**, which represented the change curve of the cross-validation error of each gene. Therefore, we selected the top four genes with the smallest cross-validation error (EIF4E, CYFIP1, IFIT5, and NUDT3); finally, we took the intersection of the three machine learning algorithms and finally obtained 3 over- lapping genes (CYFIP1, EIF4E, and IFIT5) between the three arithmetic methods (**Figure 2E**).

### Construction and evaluation of PAH diagnostic nomogram model

**Figure 3:**
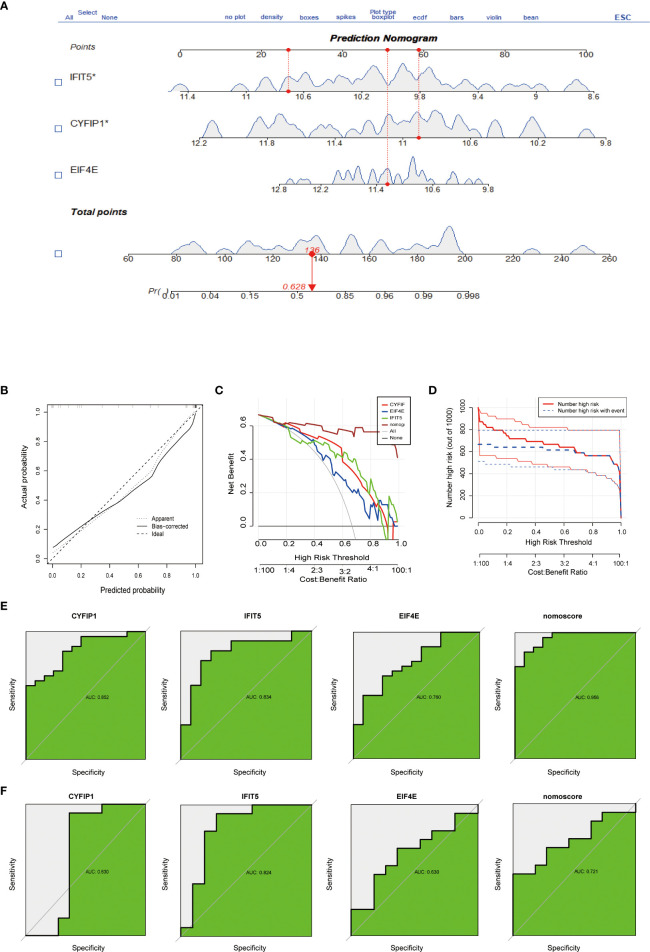
Construction and evaluation of a nomogram model for PAH diagnosis. **(A)** Nomogram model used to predict the incidence of PAH. **(B)** The calibration curve was used to assess the ability of the Nomo score model to predict. **(C)** The clinical value of the Nomo score model assessed by the decision curve. **(D)** Evaluation of the clinical impact curve of the Nomo score model based on the decision curve. **(E)** ROC curves of CYFIP1, EIF4E, IFIT5 and Nomo score in the GSE15197 dataset. **(F)** CYFIP1, EIF4E, IFIT5 and Nomo score ROC curve validation results in the GSE113439 validation set, AUC value is the area under the ROC curve.

To predict the incidence of the disease, we constructed a nomogram model for the three (CYFIP1, EIF4E, and IFIT5) disease characteristic genes according to the “Rms” software package (**Figure 3A**). The predictive ability of the nomogram model was evaluated using the calibration curve, and as shown, the nomogram model had high accuracy in predicting PAH (**Figure 3B**). Meanwhile, DCA showed that the “nomogram” curve was above the gray line, indicating that patients could benefit from the nomogram model at a high-risk threshold of 0 to 1 (**Figure 3C**). To evaluate more intuitively the clinical effect of the nomogram model, we drew the clinical impact curve based on the DCA curve, as shown in **Figure 3D**: The “high-risk number” curve and the “high-risk event number” curve are very close to the high-risk threshold curve, which demonstrated the extraordinary predictive power of the nomogram model. These results also suggested that these three genes may play a key role in the process of PAH. Finally, we further determined the diagnostic values of CYFIP1, EIF4E, and IFIT5 in the GSE15197 dataset by the area under the ROC curve and Nomo score (AUC=0.956) (**Figure 3E**). Meanwhile, to generate more accurate and reliable results, the GSE113439 dataset was adopted to verify the expression levels of 3 features, as shown in **Figure 3F**: CYFIP1 204 (AUC=0.630), EIF4E (AUC=0.824), IFIT5 (AUC=0.630) and Nomo score (AUC=0.721).

### Consensus clustering of m^7^G genes in PAH

**Figure 4:**
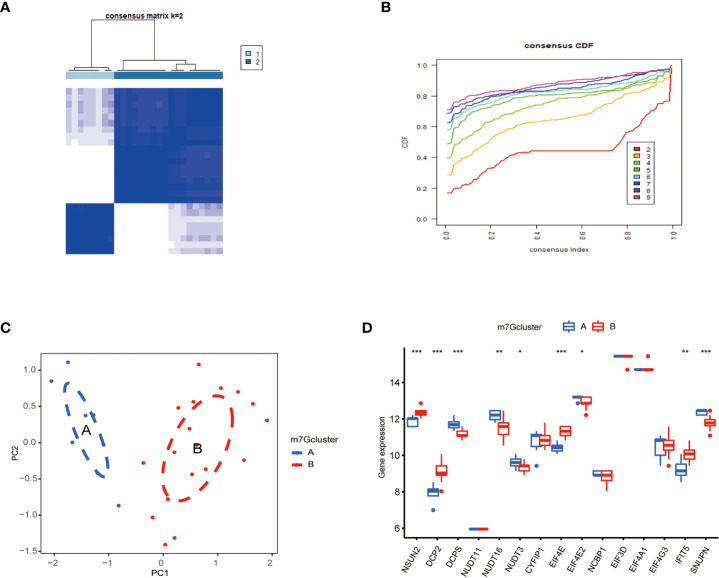
Consistent clustering of m^7^G genes. **(A)** Consistency of clustering results heatmap (k = 2). **(B)** Consensus cluster cumulative distribution function curve(k=2). **(C)** PCA analysis. Different colors represent different groups. As shown in the diagram, groups A and B are separated without any intersection. **(D)** Expression of 15 kinds of m^7^G regulators in Cluster. The abscissa represents m^7^G differential gene, and the ordinate represents the expression distribution of this related gene. *P < 0.05, **P < 0.01, and ***P < 0.001.

To classify m^7^G methylation modification patterns. We performed a consensus unsupervised cluster analysis of these 15 kinds of m^7^G regulators with significant differences using the “Consensus Cluster Plus” R software package, and the consistent cluster analysis showed that when the number of clusters K=2, PAH patients can be divided into two groups. Subgroups, which we termed m^7^G cluster A and m^7^G cluster B (**Figure 4A**). At the same time, the consensus clustering cumulative distribution function (CDF) results showed that when K=2, the grouping was the best (**Figure 4B**). Furthermore, by principal component analysis (PCA), it was observed that m^7^G cluster A and m^7^G cluster B were well differentiated (**Figure 4C**). Next, the correlations between the two subtypes determined by consensus clustering and m^7^G regulators were shown in the figure, NSUN2, DCP2, DCPS, NUDT16, NUDT3, EIF4E, EIF4E2, IFIT5, and SNUPN regulators were in m^7^G cluster A and m^7^G cluster B. There were significant differences between the two types, while NSUN2, DCP2, EIF4E, and IFIT5 were regulated in type B, and DCPS, NUDT16, NUDT3, EIF4E2, and SNUPN were down regulated in type A (**Figure 4D**).

### Correlation of biomarkers and immune cells in PAH

**Figure 5:**
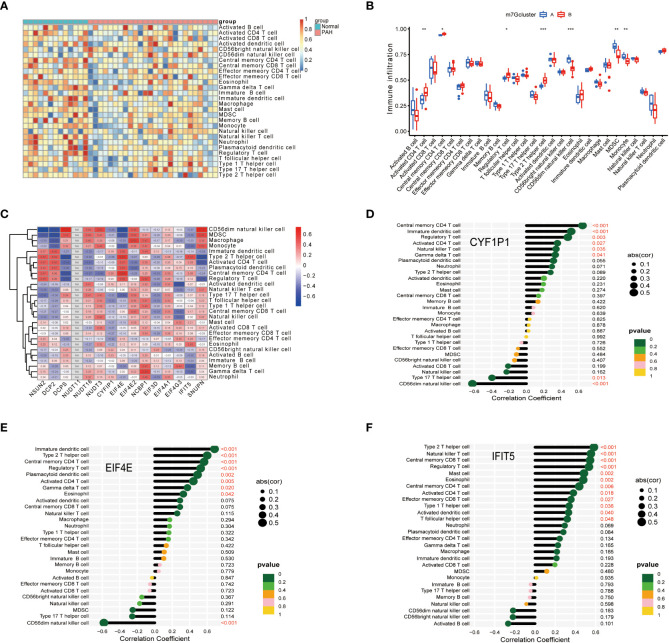
Correlation of disease characteristic genes and immune cells in PAH. **(A)** Differential analysis of immune cells. **(B)** Difference analysis of immune cells in m^7^G cluster. **(C)** Correlation analysis of immune cells and m^7^G regulators. **(D)** Correlation analysis of CYFIP1 gene and immune cells. **(E)** Correlation analysis between EIF4E gene and immune cells. **(F)** Correlation analysis between IFIT5 gene and immune cells. Spearman correlation analysis was used between genes and gene expression. The abscissa in the figure represents the correlation coefficient, the ordinate represents the immune cell, and the rightmost value represents the correlation p value, the correlation coefficient. *P < 0.05, **P < 0.01, and ***P < 0.001.

To further explore immune differences between PAH patients and normal, we used the ssGSEA algorithm to evaluate the expression of 28 immune cells in GSE15197 samples, and the results were shown in **Figure 5A**. Based on the two typing patterns of m^7^G cluster A and m^7^G cluster B, we performed a differential analysis of immune cells between the two clusters. The results showed that MDSC, monocyte, activated CD4 T cell, CD56 dim natural killer cell, regulatory T cell, and type 2 T helper cell were different in the two types. Among them, activated CD4 T cell, regulatory T cell, and type 2 T helper cell were upregulated in m^7^G cluster B, CD56 dim natural killer cell, MDSC, and monocyte were upregulated in m^7^G cluster A (**Figure 5B**). Next, we performed a correlation analysis on immune cells and m^7^G regulators, the results were shown in **Figure 5C**, red represents positive correlation, blue represents negative correlation, and the darker the color, the stronger the correlation. In addition, we also explored the correlation between biomarkers and the content of different immune cells. As shown in **Figures 5D–F**, CYFIP1 was positively correlated with central memory CD4 T cell, immature dendritic cell, regulatory T cell, activated CD4 T cell natural killer T cell, gamma delta T cell, and type 17 T helper cell, CD56 dim natural killer cell was negatively correlated; EIF4E was negatively correlated with immature dendritic cell, type 2 T helper cell, central memory CD4 T cell, regulatory T cell, plasmacytoid dendritic cell, activated CD4 T cell, gamma delta T cell, eosinophil was positively correlated, and negatively correlated with CD56 dim natural killer cell; IFIT5 was correlated with type 2 T helper cell, natural killer T cell, central memory CD8 T cell, regulatory T cell, mast cell, eosinophil, central memory CD4 T cell, activated CD4 T cell, effectors memory CD8 T cell, Type1 T helper cell, activated dendritic cell, T follicular helper cell were positively correlated.

### Consensus clustering of genes in PAH

**Figure 6:**
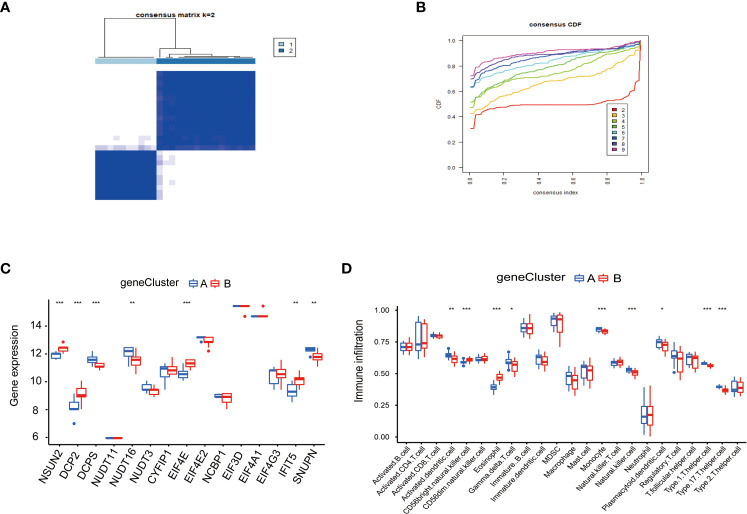
Consensus clustering of genes in PAH. **(A)** Consistency of clustering results heatmap (k=2). **(B)** Consensus cluster cumulative distribution function curve (k=2). **(C)** Expression of 15 kinds of m^7^G regulators in the gene Cluster. **(D)** Expression of immune cells in the gene Cluster. *P < 0.05, **P < 0.01, and ***P < 0.001.

To study the clinical significance of the m^7^G cluster, we screened out 2148 genes related to m^7^G phenotype in the gene set by limma package and performed unsupervised clustering analysis to divide patients into different genotypes. Consistent clustering analysis showed that when the number of clusters K=2, which the differential genes could be divided into two subgroups, which we called gene cluster A and gene cluster B (**Figure 6A**). Consensus clustering CDF results showed that grouping was optimal when K=2 (**Figure 6B**). Next, we performed a differential analysis of m^7^G and immune cells in both gene cluster A and gene cluster B. The results were shown in **Figures 6C, D**, NSUN2, DCP2, DCPS, NUDT1 6, EIF4E, IFIT5, and SNUPN regulators in these two genes Cluster. There were significant differences in the gene cluster, while NSUN2, DCP2, EIF4E, and IFIT5 were upregulated in gene Cluster A, and DCPS, NUDT16, and SNUPN were upregulated in gene Cluster B. Activated dendritic cell, CD56 bright natural killer cell, eosinophil, gamma delta T cell, monocyte, natural killer cell, plasmacytoid dendritic cell, type 1 T helper cell, and type 17 T helper cell immune cells were different between gene cluster A and gene cluster B, Activated dendritic cell, gamma delta T cell, monocyte, natural killer cell, plasmacytoid, dendritic cell, type1 T helper cell, and type 17 T helper cell immune cells were downregulated in gene cluster B, CD56 bright natural killer cell and eosinophil were down regulated in gene cluster A.

### Construction of m^7^G score

**Figure 7:**
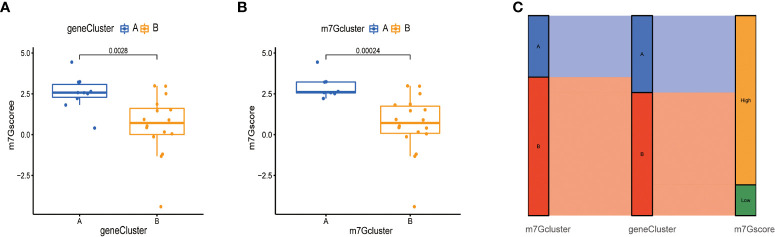
Construction of m^7^G score. **(A)** Score of m^7^G in gene Cluster. The abscissa represents different gene cluster, and the ordinate represents the score of this gene. **(B)** Score of m^7^G in m^7^G cluster. **(C)** Alluvial diagram of show gene cluster in relation to m^7^G cluster and m^7^G score.

Next, due to the individual differences and complexity of m^7^G methylation modifications, we scored the expression levels of m^7^G-related genes by the PCA method. This scoring system was named the m^7^G score. As shown in the figure, the score of m^7^G cluster A was significantly higher than that of m^7^G cluster B (**Figure 7A**), and the score of gene cluster A was significantly higher than that of gene cluster B (**Figure 7B**), indicating that m^7^G scores were different in different types. To observe the correspondence between the samples, we used the Alluvial diagram for visual analysis, and the results were shown in the figure. There was a high similarity between the results of the m^7^G cluster and the results of the gene Cluster (**Figure 7C**).

### Evaluation of marketed drugs

It was of great significance to evaluate the marketed drugs and provided new ideas for the treatment of PAH. **Table 1** demonstrated the effective drugs associated with m^7^G regulators in the DSigDB database. The top 10 drug were extracted based on p-values.

**Table 1:** Evaluation of marketed drugs.

Index	Name	P-value	Odds Ratio	Combined Score
1	Calpain inhibitor I CTD 00002578	0.002548	624.41	3729.25
2	Selenium (6+) CTD 00002696	0.002698	587.65	3476.15
3	KU-55933 CTD 00004399	0.002847	554.97	3252.89
4	Everolimus CTD 00003479	0.003596	434.22	2443.77
5	S-1,2-Dichlorovinyl-N-acetylcysteine CTD 00002159	0.003596	434.22	2443.77
6	Perhexiline CTD 00006493	0.003745	416.1	2324.86
7	Niclosamide PC3 DOWN	0.004344	356.59	1939.49
8	Zimeldine PC3 DOWN	0.004942	311.95	1656.47
9	Alexidine PC3 DOWN	0.005092	302.48	1597.17
10	Luronit CTD 00006106	0.00539	285.17	1489.48

## Discussions

In this study, we performed differential analysis by comparing lung tissue from PAH patients and normal as a result, 15 kinds of m^7^G differential molecules were screened, and 3 important diseases characteristics genes CYFIP1, EIF4E, and IFIT5 were screened by machine learning method. To predict the incidence of the disease, we constructed a nomogram model and used the ROC curve to further evaluate the accuracy of the prediction model, and the model had also been validated in the GSE113439 dataset. Furthermore, we applied the ssGSEA algorithm to detect immune cell infiltration in PAH, and the results showed that immune cell infiltration in PAH patients was significantly different from that in Normal. Next, we performed consensus clustering analysis based on 15 kinds of m^7^G differential molecules and found that there was a strong correlation between the two clusters and a variety of immune cells (activated CD4 T cell, CD56 dim natural killer cells, MDSC, monocyte, regulatory T cell, and type 2 T helper cell). We also conducted a differential analysis of the target genes CYFIP1, EIF4E, and IFIT5 in immune cells, and found that CYFIP1, EIF4E, and IFIT5 were significantly different in various immune cells. In conclusion, we investigate that CYFIP1, EIF4E, and IFIT5 may involve in the pathological progress in PAH for the first time. PAH is defined as a chronic progressive malignant pulmonary vascular disease with pathological features like cancer, such as cell proliferation, altered mitochondrial metabolism, overexpression of growth factors, etc. (42). M^7^G is associated with cardiovascular disease (19). In recent years, it has been increasingly recognized that the immune system plays an important role in the progression of PAH (43). Our study demonstrates that m^7^G regulators are closely related to a variety of immune cells.

Growing evidence suggests that T lymphocytes, dendritic cells, macrophages, monocytes, and NK cells play an important role in the immune defense of PAH (44, 45). T lymphocytes are a major component of the adaptive immune response and can be divided into helper T cells (Th cells), cytotoxic T cells (Tc cells), and regulatory T cells (Treg cells) (46). Clinical evidence suggests that Treg function is reduced in patients with IPAH (47). This study shows that activated CD4 T cell, type 2 T helper cell and regulatory T cell exhibited higher immune filtration in m^7^G cluster B, suggesting that they play an important role in the PAH process. As a key part of the innate immune system, NK cells play an important role in promoting anti-tumor immuno-therapy and controlling inflammatory and autoimmune diseases (48). *Ormiston* et al. have demonstrated through clinical evidence and experiments that NK cells play an important role in PAH angiogenesis and remodeling (49). NK cells have a protective effect on the right ventricle in rats with severe PH (48). In this study, we find a higher infiltration of CD56 dim natural killer cell in m^7^G cluster A. In addition, we demonstrate that the infiltration of MDSC and monocyte in m^7^G cluster A is higher. Evidence suggests that MDSCs and monocyte may be involved in the process of vascular remodeling and play a crucial role in the development of PAH (50). Therefore, we speculate that there may be some regulatory mode between m^7^G and the PAH immune microenvironment. At present, there is no report on the relationship between m^7^G and the PAH immune microenvironment. Our study will provide preliminary insights into the immune infiltration pattern of PAH and its underlying immune modulator mechanisms. Simultaneously, we also analyzed the correlation between disease signature genes and immune cells. We identify CYFIP1, EIF4E, and IFIT5 as key genes of PAH by machine learning method. Among these genes, the researchers have found that CYFIP1 plays an important role in brain functional connectivity and corpus callous function, suggesting that copy number variation in the human CYFIP1 gene is associated with autism spectrum disorder and schizophrenia (51, 52). Meanwhile, because CYFIP1 expression decreases during epithelial tumor invasion, CYFIP1 has been proposed as an invasion suppressor in epithelial cancers (53). At present, there is no report on CYFIP1 and the immune microenvironment of PAH. This study shows that CYFIP1 is found in central memory CD4 T cell, immature dendritic cell, regulatory T cell, activated CD4 T cell, natural killer T cell, and gamma delta T cell. There are significant differences in 17 T helper cell, and CD56 dim natural killer cell. Therefore, our study is the first time to link CYFIP1 with the immune microenvironment of PAH, which may provide a new way for CYFIP1 function research. Substantial evidence suggests that dysregulated expression of EIF4E is associated with 30% of human tumors, including head and neck cancer (54), endometrial cancer (55) and prostate cancer (56). This improves understanding of the role of EIF4E in cancer biology. Our research has shown that EIF4E is in an immature dendritic cell, type 2 T helper cell, central memory CD4 T cell, regulatory T cell, plasmacytoid dendritic cell, activated CD4 T cell, gamma delta T cell, eosinophil CD56 dim natural killer cells have significant differences. Therefore, we speculate that EIF4E plays an important role in the immune microenvironment of PAH. IFIT5 is a member of the interferon-induced tetrapeptide repeat family (57). Its expression is positively correlated with pathological features of bladder cancer and predicts poor prognosis in BCA patients (58). At the same time, IFIT5 mRNA levels are significantly elevated in high-grade prostate cancer (59). This study has investigated that IFIT5 can be found in type 2 T helper cell, natural killer T cell, central memory CD4 T cell, regulatory T cell, mast cell, eosinophil, and central memory CD8 T cell. There are significant differences in CD4 T cell, CD8 T cell, type 1 T helper cell, activated dendritic cell, and T follicular helper cell, which provide evidence for the involvement of m^7^G in the regulation of the PAH immune microenvironment. However, the correlation between m^7^G and immune cells still requires further study. At present, the regulation of RNA methylation in diseases has been studied with m^6^A and m^5^C. M^5^C methylation can regulate tumor microenvironment infiltration characterization of lung adenocarcinoma and immune microenvironment of multiple myeloma (60, 61). Interestingly, the m^6^A modulator may be a promising biomarker for the diagnosis and treatment of PAH in the monocrotaline-induced pulmonary hypertension model of rats (62–64). Currently, there is very little research on the effect of m^7^G methylation on PAH. Previous studies have shown that lncRNAs are significantly upregulated compared with non-m^7^G lncRNAs in HPH rats (23). The clinical significance of the results of this study is to find that further intervention of m^7^G may be of great significance as a new strategy to combat the occurrence and development of PAH. These findings are helpful to further understand the pathogenesis of PAH and provide a new target for the treatment of PH.

Overall, this study constructs a model that can predict the incidence of PAH disease from the perspective of m^7^G methylation. And the underlying mechanism of m^7^G methylation modification in PAH is related to the immune microenvironment. Meanwhile, our study has some limitations, considering the individual heterogeneity of PAH. Our findings should be further validated using more multicenter clinical data. In the future, it will be necessary to collect in-house clinical data to experimentally validate some of the findings in this study. For example, whether m^7^G regulates inflammatory factors and thus, participates in the progression of PAH.

## Conclusions

In conclusion, this study provides more information toward understanding the pathophysiological mechanism of PAH by starting from the broad regulatory mechanism of m^7^G methylation modification on the PAH immune microenvironment. Our study has identified CYFIP1, EIF4E, and IFIT5 as potential novel pharmacology biomarkers in PAH. Furthermore, their association with immune cell infiltration may facilitate the development of immune therapy in PAH.

## Data availability statement

The datasets presented in this study can be found in online repositories. The names of the repository/repositories and accession number(s) can be found in the article/supplementary material.

## Author contributions

DW, YM, and YB conceived the original scope of this manuscript. DW and YM designed the experiments and manuscript, and figures. YB and DZ critically reviewed the final manuscript. All authors contributed to the article and approved the submitted version.

## Funding

This work is supported by grants from the National Nature Science Foundation of China No.81803530.

## Acknowledgments

We acknowledge the GEO database for providing the platforms, and contributors for uploading meaningful datasets.

## Conflict of interest

The authors declare that the research is conducted in the absence of any commercial or financial relationships that can be construed as a potential conflict of interest.

## Publisher’s note

All claims expressed in this article are solely those of the authors and do not necessarily represent those of their affiliated organizations, or those of the publisher, the editors and the reviewers. Any product that may be evaluated in this article, or claim that may be made by its manufacturer, is not guaranteed or endorsed by the publisher.

## Author disclaimer

This is a computationally-based study that needs to be verified experimentally.

## Abbreviations

PAH, pulmonary arterial hypertension; PVR, pulmonary vascular resistance; PH, pulmonary hypertension; IPAH, Idiopathic pulmonary arterial hypertension; MCT, monocrotaline; METTL1, methyltransferase like 1; m^6^A, N6-methyladeno-sine; m^5^C, 5-methylcytosine; m^7^G, 7-methylguanosine; lncRNAs, long noncoding RNAs; DSigDB, Drug Signatures Database; LASSO, Least Absolute Shrinkage and Selection Operator; RF, random forest; SVM-RFE, support vector machine recursive feature elimination; ROC, receiver operating characteristic; ssGSEA, single-sample gene set enrichment analysis; DCA, decision curve analysis; CDF, cumulative distribution function; PCA, principal component analysis; HPH, hypoxic pulmonary hypertension.
